# Intravitreal aflibercept for choroidal neovascularization secondary to angioid streaks in a non-responder to intravitreal ranibizumab

**DOI:** 10.2147/IMCRJ.S166473

**Published:** 2018-09-18

**Authors:** Olga E Makri, Foteini N Tsapardoni, Panagiotis Plotas, Athina Pallikari, Constantine D Georgakopoulos

**Affiliations:** Department of Ophthalmology, University of Patras, Patras, Greece, cgeorg@upatras.gr

**Keywords:** aflibercept, ranibizumab, angioid streaks, choroidal neovascularization, intravitreal

## Abstract

**Purpose:**

To report the 12-month outcomes of a patient switching from intravitreal ranibizumab to aflibercept for choroidal neovascularization (CNV) associated with angioid streaks (AS).

**Results:**

A 42-year-old Caucasian female with CNV associated with AS underwent intensive treatment with ranibizumab without significant functional or anatomic change. Treatment was then switched to aflibercept and the patient received the proposed age-related macular degeneration treatment regimen. After 3 loading doses of aflibercept, best-corrected visual acuity (BCVA) improved from 3/10 to 6/10, while optical coherence tomography (OCT) demonstrated resolution of the subretinal fluid with a reduction of the intraretinal fluid. After 12 months and 7 intravitreal injections of aflibercept, BCVA returned to 3/10, while OCT had demonstrated further morphologic improvement.

**Conclusion:**

Our case shows that aflibercept may be an alternative treatment for advanced cases of CNV associated with AS that respond insufficiently to ranibizumab injections. Prospective studies are required to further evaluate the effect of aflibercept and to propose a standardized treatment protocol for this entity.

## Introduction

Angioid streaks (AS) are linear cracks of the abnormally calcified and fragile Bruch’s membrane that spread radially outward from the peripapillary area. They may be idiopathic or a manifestation of several systemic disorders, such as pseudoxanthoma elasticum, Ehlers–Danlos syndrome, Paget’s disease, and hemoglobinopathies.^1^ A common, challenging, and sight-threatening complication of AS is choroidal neovascularization (CNV). The efficacy of anti-vascular endothelial growth factor (VEGF) agents, such as bevacizumab or ranibizumab, in the treatment of AS-associated CNV has been evaluated, with ranibizumab recently receiving official approval for this condition.^2^–^4^ However, information regarding the treatment of AS-associated CNV with aflibercept is limited.^5^–^7^ Early, yet encouraging, clinical data of switching from ranibizumab to aflibercept for the treatment of AS-associated CNV have been reported by Esen et al.^5^ We report the 1-year results of treatment with aflibercept for AS-associated CNV after switching from ranibizumab. Written informed consent was obtained from the patient in order for this case report (including any case details and accompanying images) to be published.

## Case report

**Figure 1:**
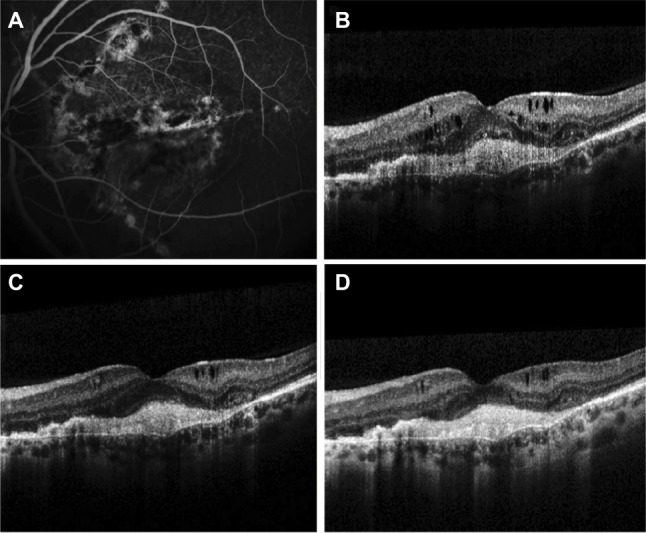
Outcomes at 13 months after ranibizumab treatment **(A, B)**, after 3 loading doses of aflibercept **(C)** and after 1 year of aflibercept treatment **(D)**. **Notes: (A)** Thirteen months after treatment with ranibizumab, fluorescein angiography depicts AS around the optic disc and leakage of the dye originating from the CNV, while staining of the fibrotic element of the lesion in the fovea is observed. **(B)** OCT scan at that point demonstrates subfoveal hyper-reflective tissue associated with CNV and subretinal fibrosis between Bruch’s membrane and the retinal pigment epithelium. Intraretinal fluid and cysts and a subtle accumulation of subretinal fluid are also noted. The CFT is 426 μm. **(C)** The OCT scan following the 3 loading doses of aflibercept reveals resolution of the subretinal fluid with absorption of the intraretinal fluid and significant reduction of the remaining intraretinal cysts. The CFT is reduced to 348 μm. **(D)** After 1 year of treatment with 7 IVIs of aflibercept, OCT demonstrates further reduction of the intraretinal cysts while the subretinal fibrosis persists. The CFT is 325 μm. **Abbreviations:** AS, angioid streaks; CFT, central foveal thickness; CNV, choroidal neovascularization; IVI, intravitreal injection; OCT, optical coherence tomography.

A 42-year-old Caucasian female with pseudoxanthoma elasticum, who had been diagnosed with bilateral AS and CNV secondary to AS in the left eye (OS), was treated with 12 intravitreal injections (IVIs) of ranibizumab (0.5 mg [50 µL]) over a period of 13 months. Despite the intensive treatment with ranibizumab, no significant functional or anatomic change was observed. One month after the last administration of ranibizumab, best-corrected visual acuity (BCVA) was 10/10 in right eye (OD) and remained stable (3/10) in OS. Fundoscopy demonstrated peau d’orange fundus appearance and AS as multiple irregular linear branching subretinal streaks, emanating radially from the optic disc without sparing the fovea. An elevated gray-yellow subretinal lesion compatible with CNV was observed in the fovea in OS, adjacent to a large fibrotic lesion. Fluorescein angiography clearly showed streaks around the optic disc and leakage of the dye originating from the CNV, while staining of the fibrotic element of the foveal lesion was observed (Figure 1A). Optical coherence tomography (OCT) imaging revealed signs of active CNV in OS with intraretinal and subretinal fluid accumulation (Figure 1B).

It was at that point a switch of treatment to aflibercept was agreed (administered by IVI), using the proposed treatment regimen for age-related macular degeneration (AMD). Following the signing of an informed consent form, the patient received a loading dose consisting of 3 consecutive IVIs of aflibercept (2 mg [50 µL]) monthly, followed by bimonthly aflibercept administration at the same dose. The 3 loading doses of aflibercept led to an improved BCVA of 6/10 in OS, while OCT demonstrated resolution of the subretinal fluid with reduction of the intraretinal fluid (Figure 1C). Two months after the third dose of aflibercept, BCVA decreased to 3/10 and ceased to improve thereafter. After a 12-month treatment period and 7 IVIs of aflibercept, BCVA remained at 3/10 in OS, while OCT demonstrated further morphologic improvement as indicated by reduction of the intraretinal fluid (Figure 1D).

## Discussion

Aflibercept is a recombinant fusion protein that binds with a high affinity and disables all VEGF isoforms, including all isoforms of VEGF-A and VEGF-B, as well as placental growth factor (PGF). It has been approved for the treatment of neovascular AMD, diabetic macular edema, macular edema due to retinal vein occlusion, and myopic CNV.^8^ However, information on the long-term effect of aflibercept on AS-associated CNV is currently limited to a few isolated case reports. In 2 treatment-naïve patients, prompt intervention and administration of 1 and 3 IVIs of aflibercept (pro re nata) resulted in a favorable outcome that was maintained over a period of 12 and 9 months, respectively.^6^ Administration of aflibercept in a treat-and-extend regimen resulted in complete restoration of BCVA in another treatment-naïve patient with early stage disease who received 6 IVIs of aflibercept over a period of 1 year.^7^ Aflibercept has also been used as first line treatment in a recently published case of polypoidal choroidal vasculopathy secondary to AS. A treatment-naïve eye was administered aflibercept using the treat-and-extend regimen, with prompt and complete regression of the polypoidal lesion. This particular eye was then followed up and treated with 15 injections of aflibercept over a period of 33 months. The initial response, with complete restoration of BCVA, was maintained throughout the study period, while the treatment intervals were extended.^9^

Although ranibizumab is an approved treatment for AS-associated CNV,^4^ isolated case reports indicate that a switch to aflibercept may be an effective alternative in patients with persistent AS-associated CNV who respond insufficiently to ranibizumab.^5^ Aflibercept and ranibizumab are two different molecules, and in vitro studies have demonstrated a greater binding affinity for VEGF-A with aflibercept compared with ranibizumab.^10^ The assumption of this potential difference in mechanism of action and effect may establish this rationale of switching to aflibercept as an alternative treatment for ranibizumab-resistant AS-associated CNV.^5^,^9^ Esen et al^5^ were the first to report the beneficial effect of switching from ranibizumab to aflibercept in a patient with refractory CNV secondary to AS. A loading dose of 3 monthly IVIs of aflibercept resulted in a satisfactory response; however, long-term results were not reported. Long-term favorable results of treatment with aflibercept in a treat-and-extend protocol have been reported in 2 eyes with polypoidal choroidal vasculopathy secondary to AS that have previously undergone ranibizumab treatment.^9^

In our case, persistent lesion activity, despite high retreatment frequency with ranibizumab, was an indication for switching to aflibercept. The aflibercept treatment protocol applied was suggested for the treatment of neovascular AMD.^8^ Although the patient showed poor prognostic factors, such as long-standing, ranibizumab-resistant CNV secondary to AS and established subretinal fibrosis, the functional and anatomic response was apparent during the loading dose phase of aflibercept treatment. However, BCVA returned to baseline values after bimonthly administration of aflibercept. More intensive treatment with aflibercept, such as fixed monthly administration throughout the treatment period, may have been a more appropriate choice for this patient with preservation of the initially observed functional response.

## Conclusion

To conclude, AS-associated CNV is a potential vision-threatening entity. Although ranibizumab is the officially approved anti-VEGF agent, aflibercept may be a promising alternative for the management of advanced cases of refractory AS-related CNV that have responded insufficiently to prior ranibizumab injections. However, due to the rarity of this entity, this reported experience is limited to the particular case report. Prospective studies with a large cohort of patients are required to further evaluate the effect of aflibercept on CNV due to AS and to propose a standardized treatment protocol for this clinical entity.
